# Editorial: Nutrition for an aging brain

**DOI:** 10.3389/fnut.2024.1405643

**Published:** 2024-05-29

**Authors:** Elżbieta Wieczorek, David Vauzour, Matthew G. Pontifex, Domenico Nuzzo

**Affiliations:** ^1^Department of Biochemistry, Molecular Biology and Biotechnology, Faculty of Chemistry, Wrocław University of Science and Technology, Wrocław, Poland; ^2^Norwich Medical School, Faculty of Medicine and Health Sciences, University of East Anglia, Norwich, United Kingdom; ^3^Institute for Biomedical Research and Innovation, National Research Council of Italy, Palermo, Italy

**Keywords:** brain, Mediterranean diet, lifestyle, ultra-processed foods, nutrition

Neurological disorders stand as the primary cause of both physical and cognitive disabilities worldwide, impacting ~15% of the global population. Over the past three decades, the absolute number of affected individuals has seen a significant increase (1). Moreover, the burden of chronic neurodegenerative conditions is projected to at least double in the next two decades. This trajectory, largely fueled by the expanding elderly demographic, presents an immense challenge to maintaining accessible neurological care for all. Recognizing these impending challenges, esteemed institutions such as the World Health Organization have raised the alarm, noting that existing and current resources allocated to neurological services fall short of meeting the global demand, thus rendering current neurological care models unsustainable and necessitating a comprehensive redesign (2). As the search to find effective therapies continues, growing evidence suggests that nutrition may play a crucial role in the management and prevention of these highly debilitating conditions. A diet that includes a variety of fresh foods rich in food bioactives, vitamins, minerals, and omega-3 fatty acids can help protect the brain and slow the progress of neurodegenerative diseases (3, 4). For example, polyphenols and vitamins are known for their roles in protecting brain cells from inflammation and oxidative stress, both of which have been implicated in the development of neurodegenerative diseases. Colorful fruits and vegetables, such as berries, spinach, and tomatoes, are rich in antioxidants such as vitamins A, C, and E, which may play a crucial role in protecting brain cells. The study conducted by Liu et al., using a relatively large sample from the United States, suggests that elevated intake of iron and copper from food is correlated with an elevated risk of Parkinson's disease. Conversely, increased consumption of vitamin C, vitamin K, and zinc from dietary sources is associated with a decreased risk of developing Parkinson's disease (Liu et al.). On the other side, protein malnutrition appears to pose a potential risk for senile dementia, with inadequate intake correlating with early cognitive decline. Conversely, sufficient protein intake in older adults is linked to improved memory function and a reduced risk of cognitive impairment. Given the role of diet as a modifiable factor in cognitive decline, extensive research has investigated various dietary approaches to prevent dementia onset in older individuals. However, conclusive evidence remains scarce. Polis and Samson's review further adds to our understanding of effective interventions aimed at enhancing cognitive wellbeing in aging populations.

The Mediterranean diet, characterized by an abundance of fruit, vegetables, whole grains, legumes, fish, and olive oil, along with a healthy lifestyle (physical and mental activity, stress reduction, and social interaction), has been associated with a reduction in the risk of developing neurodegenerative diseases (Figure 1).

**Figure 1:**
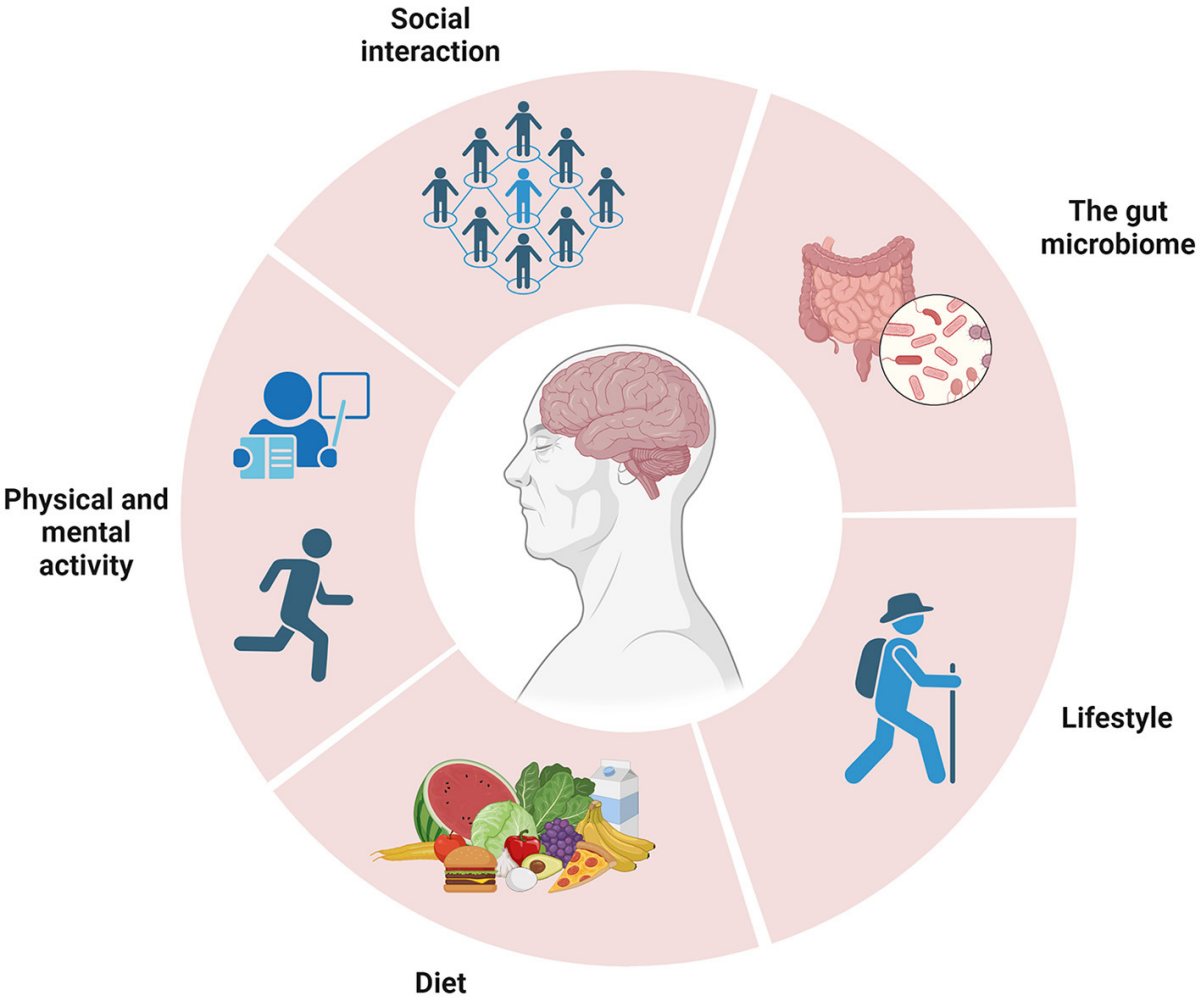
Schematic representation of neurodegeneration characterized by various components. The Mediterranean diet is renowned for its health benefits, including a reduced risk of brain disease and promoting overall wellbeing and longevity. Image created with BioRender.com.

This regimen provides a wide range of beneficial nutrients for the brain and is notable for its potential to help maintain cognitive health and reduce neurological decline (Arora et al.). However, relying solely on dietary intake is often not sufficient to provide the necessary quantities of essential bioactive compounds to exert a protective effect on the brain. This deficiency highlights the need to explore integrated nutritional strategies, incorporating supplementation with specific elements, to effectively combat neurodegenerative diseases. This perspective highlights the importance of identifying key bioactive compounds and developing effective means to integrate them into daily dietary regimens, thus offering further potential avenues for the management and prevention of these serious neurological conditions (Aguree et al.; Hao et al.).

Conversely, ultra-processed foods, which often contain high amounts of added sugars, saturated fats, salt, and artificial additives, have come under increasing scrutiny for their potential negative health effects. While the direct connection between the consumption of ultra-processed foods and neurodegenerative diseases is still being studied, there is evidence to suggest a possible correlation. In fact, ultra-processed foods can promote oxidative stress and chronic inflammation. Inflammation has been linked to many neurodegenerative diseases, including Alzheimer's disease and Parkinson's disease. Importantly, research into the specific effects of ultra-processed foods on the brain is still ongoing, and further studies are needed to fully understand the relationship between these foods and neurodegenerative diseases. However, current evidence suggests that limiting the consumption of ultra-processed foods and opting for a diet rich in whole foods, such as fruits, vegetables, whole grains, and lean proteins, may help support brain health and reduce the risk of these serious neurological conditions. Promoting greater awareness of the potential risks associated with ultra-processed foods could play a crucial role in preventing neurodegenerative diseases and improving the general health of the population (Claudino et al.).

Ultimately, in this fusion of nutrition and neuroscience, we envision a future in which dietary tactics evolve beyond basic sustenance, emerging as crucial elements within comprehensive approaches to brain wellness. The prospect of accessible, personalized, and scientifically supported interventions instills optimism despite the growing obstacles presented by neurodegenerative conditions. Advances in scientific research offer the prospect of a more solid understanding of cognitive decline and nutrition.

## Author contributions

EW: Writing – original draft, Writing – review & editing. DV: Writing – original draft, Writing – review & editing. MP: Writing – original draft, Writing – review & editing. DN: Writing – original draft, Writing – review & editing.
